# Family Caregiver Burden in the Context of the Long-Term Care Insurance System

**DOI:** 10.2188/jea.14.139

**Published:** 2005-03-18

**Authors:** Yumiko Arai

**Affiliations:** 1 Research Unit for Nursing Caring Sciences and Psychology, National Institute for Longevity Sciences (NILS).

**Keywords:** burden, caregiver, Japan, long-term care

## Abstract

This paper covers our recent work regarding family caregiver burden for elderly. The topics are as follows: cross-sectional studies on caregiver burden; changes in caregiver burden; appropriateness of the Long-Term Care insurance assessment scheme; attitude towards caregiving among caregivers; and the development of the short version of the Japanese version of the Zarit Caregiver Burden Interview (J-ZBI_8).

## Introduction

An increase in the number of impaired elderly people and a concomitant decrease in the capacity of informal care (partly due to the increasing development of the nuclear family and more career-oriented women) have now made caregivers’ burden a social issue not only in Japan but many in developed countries.

It was Professor Steven Zarit of Pennsylvania State University that first proposed an operational definition of caregiver burden as the extent to which caregivers perceived their emotional or physical health, social life and financial status as suffering as a result of caring for their relative. He then developed an assessment tool for the feelings of caregiver burden based on the above definition, the Zarit Burden Interview (ZBI).^1^^,^^2^ The ZBI is now the instrument most widely used in North America and Europe for assessing the burden experienced by family caregivers who look after the community-residing impaired elderly.

We developed a Japanese version of this assessment scheme, called J-ZBI,^3^ which is currently the most widely used assessment tool for caregiver burden in Japan. This paper is a review of our most recent work related to caregiver burden.

## Cross-sectional studies on caregiver burden

A study was conducted using the J-ZBI in Japan in 1998 in order to identify the factors related to the feelings of burden experienced by family caregivers who looked after the impaired elderly. As in previous studies in North America and Europe, it was found that behavioural disturbances were a strong correlate of the feelings of caregiver burden (odds ratio = 4.75, 95% confidence interval = 1.45-15.54, p=0.01).^4^ The above findings did not differ after the Long-Term Care (LTC) insurance system was implemented; behavioural disturbances have remained a strong correlate of the feelings of caregiver burden (odds ratio = 7.16, 95% confidence interval = 1.48-34.70, p=0.01).^5^

## Changes in caregiver burden

We conducted a survey every year from 1998 through 2001 targeting all disabled elderly and their principal caregivers residing in Matsuyama Town located in rural northern Japan. The design of this Matsuyama Caregiver study was described in detail elsewhere.^6^^,^^7^

**Figure 1.**
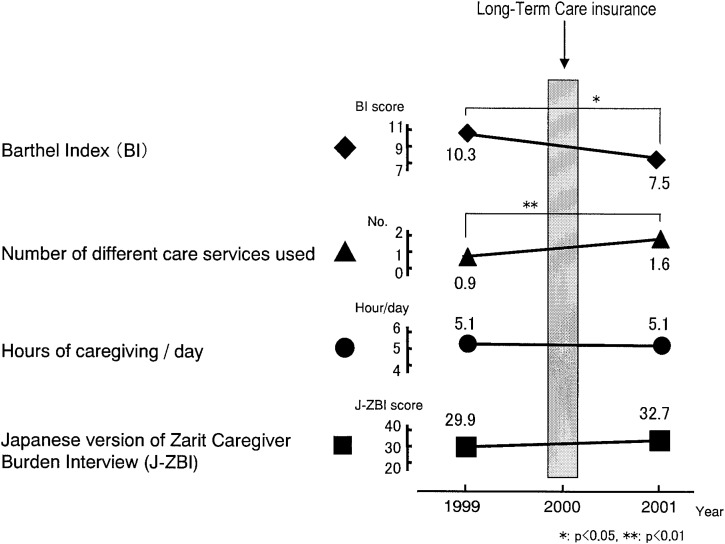
Changes in variables between 1999-2001.

As a part of the study, a longitudinal analysis was conducted between October 1998 and October 2000. This analysis was an attempt to determine how caregiver burden may have changed before and after the implementation of the LTC insurance system. It was found that the number of services used in 2000 was significantly greater than in 1998. However, caregiver burden itself did not change from 1998 to 2000, the first year in which the new system had been in place.^8^ We conducted a similar analysis to compare caregiver burden between 1999 and 2001. As shown in Figure 1, there was no significant difference between the mean J-ZBI score in 1999 and 2001.^9^ Overall, these longitudinal studies show that the degree of caregiver burden did not change among the caregivers who had been providing care prior to the launch of the LTC insurance scheme.

**Figure 2.**
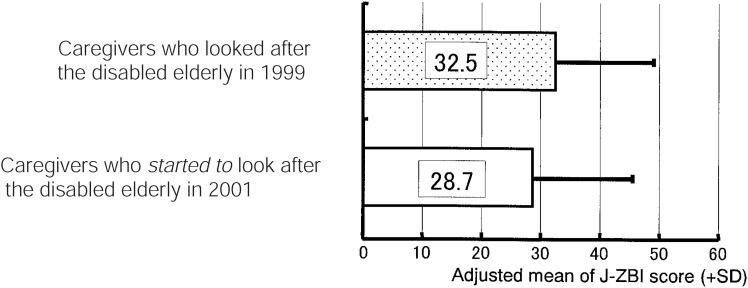
Comparisons of J-ZBI score between caregivers who looked after the disabled elderly in 1999 and those who *started to* look after the disabled elderly in 2001. Adjusted by caregivers’ age, caregivers’ sex (female=1), age of disabled elderly, duration of caregiving(month), no. of family members, ADL score (Barthel Index), score of behavioral disturbances (TBS).

We also made comparisons between caregivers of the disabled elderly in 1999 and those entrusted with their care in 2001 in terms of their degree of caregiver burden by Analysis of Co-variance (ANCOVA), adjusting for other variables. As shown in Figure 2, the adjusted J-ZBI mean score in 2001 was not significantly different from that in 1999, indicating that feelings of burden among caregivers did not change after the implementation of the LTC insurance system.^9^

## Appropriateness of LTC insurance assessment scheme

**Figure 3.**
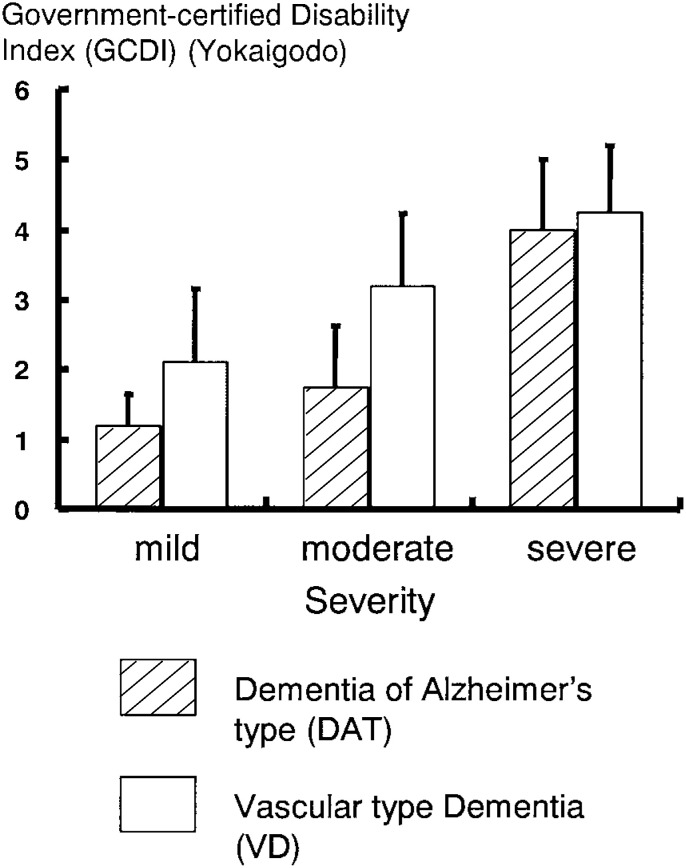
Government-certified Disability Index(GCDI) (Yokaigodo) and severity in DAT and VD patients.

In the LTC insurance, services are allocated based on the Government-certified Disability Index (GCDI) (Yokaigodo).^10^ We were interested in whether the LTC insurance system in Japan indeed developed a fair and appropriate way of allocating resources to the nation’s disabled elderly population, especially those with dementia. Specifically, our study investigated whether the GCDI scores under the LTC insurance program adequately reflected the needs of people with DAT (dementia of Alzheimer’s type) and VD (vascular-type dementia). As a result, the GCDI score among the DAT patients proved to be lower than among the VD patients, indicating that DAT patients were classified as “less disabled” on their GCDI than VD patients, as shown in Figure 3.^11^ Since the amount of care services patients are allowed to use under the LTC insurance plan is determined solely by the GCDI score, it appears that the people with DAT in the study were allowed fewer care services despite the fact that the severity of their dementia was the same as for a VD patient.

## Attitude toward caregiving among caregivers

The LTC system has demonstrably changed the attitudes of caregivers. It was found that more caregivers came to believe that society must look after the elderly after only one year under the new program.^12^ In the short space of a year, there was an obvious shift from the idea that the care of old folks falls to the family to the virtually unheard-of notion that society must shoulder the problems of the world’s fastest-graying population.^12^^,^^13^

## Development of short version of Japanese version of Zarit Caregiver Burden Interview (J-ZBI_8): its reliability and validity

In the era of LTC insurance, it has become even more important to monitor the well-being of not only the impaired elderly but also the family caregivers. In this regard, in order to facilitate the assessment of family caregiver burden in clinical settings, we proposed a short version of the J-ZBI, consisting of the following two factors: Personal strain (5 items) and Role strain (3 items). These eight items are presented in Table 1. It was demonstrated that the newly proposed short version, J-ZBI_8, had high reliability, concurrent validity and construct validity.^14^ Subsequently, the cross validation was conducted.^15^^,^^16^ Overall, the J-ZBI_8 produced results comparable to those of the full version, i.e., the J-ZBI. The shorter yet no less reliable and valid eight-item version will thus lead to easier administration of the instrument for assessing family caregiver burden in clinical settings.

**Table 1.** The short version of the Japanese version of the Zarit Caregiver Burden Interview (J-ZBI_8).^14^^-^^16^

For each question, chose one of the following answers:
0. Never 1. Rarely 2. Sometimes 3. Quite frequently 4. Nearly always

Questions
1. Do you feel embarrassed over your relative’s behavior? *
2. Do you feel angry when you are around your relative? *
3. Do you feel that your relative currently affects your relationship with other family members or friends in a negative way? ^†^
4. Do you feel strained when you are around your relative? *
5. Do you feel that your social life has suffered because you are caring for your relative? ^†^
6. Do you feel uncomfortable about having friends over because of your relative? ^†^
7. Do you wish you could just leave the care of your relative to someone else? *
8. Do you feel uncertain about what to do about your relative? *


*: J-ZBI_8 Personal Strain

^†^ : J-ZBI_8 Role Strain
